# Supplementary material for: The impact of antimalarial resistance on the genetic structure of Plasmodium falciparum in the DRC
Source: Nat Commun. 2020 Apr 30;11:2107. doi: 10.1038/s41467-020-15779-8 (PMC7192906; doi:10.1038/s41467-020-15779-8)
Supplement: Supplementary file 1 — Supplementary Information [file 41467_2020_15779_MOESM1_ESM.pdf]

SUPPLEMENTARY INFORMATION

Supplementary information for the paper: Verity et al. *The Impact of Antimalarial Resistance on the Genetic Structure of Plasmodium falciparum in the DRC*

TABLE OF CONTENTS

1. Supplementary Note 1 Page 2
2. Supplementary Note 2 Page 8
3. Supplementary Table 1 Page 16
4. Supplementary Figure 1 Page 17
5. Supplementary Figure 2 Page 18
6. Supplementary Figure 3 Page 19
7. Supplementary Figure 4 Page 20
8. Supplementary Figure 5 Page 21
9. Supplementary Figure 6 Page 22
10. Supplementary Figure 7 Page 23
11. Supplementary Figure 8 Page 24
12. Supplementary Figure 9 Page 25
13. Supplementary Figure 10 Page 26
14. Supplementary Figure 11 Page 27
15. Supplementary Figure 12 Page 28
16. Supplementary Figure 13 Page 29
17. Supplementary Figure 14 Page 30
18. Supplementary Figure 15 Page 31
19. Supplementary References Page 32

Supplementary Note 1

Supplementary Methods

MIP Design: We used two distinct MIP panels - a genome-wide panel designed to capture overall levels of differentiation and relatedness, and a drug resistance panel aimed at polymorphic sites known to be associated with antimalarial resistance. The drug resistance MIP panel included mutations in genes atp6 (L263E, E431K, A623E, S769N), crt (C72S, M74I, N75E, K76T, H97L, H97Y, A220S, N326S, I356T), cytb (M133I, Y268S, Y268C, V284K), dhfr-ts (A16V, N51I, C59R, S108T, I164L, T185), dhps (S436A, A437G, K540E, A581G, A613S), kelch13 (S436A, A437G, K540E, A581G, A613S), mdr1(N86Y, Y184F, S1034C, N1042D, D1246Y), mdr2 (T484I). In addition the following putative drug resistance mutations were included in the drug resistance panel: pib7 (C1484F), pph (V1157L), fd (D193Y), PF3D7_1322700 (T236I), PF3D7_1451200 (N71N) and arps10 (V127M)[1]. When selecting targets for the genome-wide panel, we used publicly available *Plasmodium falciparum* whole genome sequences provided by the Pf3k project (Data Release 5) and *P. falciparum* Community project (Data Release 4), which are part of the wider MalariaGEN Consortium[2,32]. This consisted of 923 samples in total, from Cameroon (n=134), the Democratic Republic of the Congo (n=285), Kenya (n=52), Malawi (n=369), Nigeria (n=5), Tanzania (n=66), and Uganda (n=12) (**Supplementary Data 1**). The genomic sequence from these samples underwent alignment, variant calling, and variant-filtering following the Pf3k strategy consistent with the Genome Analysis Toolkit (GATK, version 3.6) Best Practices with minor modifications[3–6]. Reads were aligned to the *P. falciparum* 3d7 reference assembly genome (version 3) using BWA-MEM with a raised base-match bonus (A=2) and clip penalty for local alignment (L=15) for increased sensitivity and specificity through hypervariable regions, and with all other flags set to default[7,8]. Given that there were paired-end reads, we used samtools fixmates, to synchronize any overlapping paired-end bases[9]. Mate-fixed reads were then deduplicated and merged with Picard Tools (version 2.2.4), MarkDuplicates and MergeSamFile respectively[10]. Finally, we performed local realignment of complex regions using GATKIndelRealigner

Following best practices for variant calling, we first used the GATK BaseRecalibrator tool to adjust our samples' base-quality scores using the sequences from the *P. falciparum* Genetic Cross project as the training set. Variant discovery was performed separately on each recalibrated binary alignment map (BAM) file using GATK HaplotypeCaller with the minimum

Phred score for a variant to be called at 30 and a ploidy of one. Setting the ploidy to one shifts the genotype-call to the major haplotype in polyclonal infections, in expectation. The individual variant call files (VCFs) then underwent joint variant discovery with the GATK GenotypeGVCFs tool. Following this step, we used the GATK VariantRecalibrator and ApplyRecalibration tools to recalibrate our discovered single-nucleotide-polymorphisms (SNPs) and insertion-deletions (INDELs). For SNP recalibration tuning, we again used the *P. falciparum* genetic crosses as the training set with the quality depth, mapping quality, fisher-score, strand-odds ratio, and allele depth as the covariate considered and the maximum gaussian clusters set to eight processes. Similarly, for INDEL recalibration, the *P. falciparum* cross data was again used to train the model but only quality depth, fisher-score, strand-odds ratio, and allele depth were considered as covariates and the maximum gaussian clusters were set to four processes. In the ApplyRecalibration step, the truth sensitivity level for filtering was set at 99% for both SNPs and INDELs. Finally, from the recalibrated-joint VCF, we filtered all variants with a variant-quality recalibration log-odds of less-than or equal to zero. In addition, variants were excluded if they were not within the “core” genome as defined by the Pf3k project.

To further decrease our false discovery rate, we subsetted to biallelic SNPs and excluded all samples (original BAMs) with fewer than 70% of loci that were callable as determined by GATK CallableLoci with flags set to a minimum base quality of 20, minimum mapping quality of 10, and a minimum depth of 4. In addition, we excluded samples from Uganda and Nigeria, as these countries did not have enough high-quality genomes for analysis. Next, we separated the joint-VCF into country-level VCF and excluded intervals that had fewer than 5-fold coverage at 50% of loci within a given country using GATK CoveredByNSamplesSites (version 3.4.46). The country-level VCFs were then re-merged and annotated using snpEFF and the Pf3D7v91 genome pre-package in the snpEFF databases.

From this filtered-VCF, we calculated Weir and Cochram’s F_{ST} with respect to country for each biallelic locus[11]. The 1,000 loci with the highest F_{ST} values were considered for MIP design as phylogeographically informative loci. Of these 1,000 potential loci, 739 were identified as regions that were suitable for MIP-probe design. Separately, from the combined SNP file, we identified 1,595 potential loci that had a minor-allele frequency greater than 5%, had an F_{ST} value between 0.005 and 0.2, and were annotated by snpEFF (version 4.3s) as functionally silent mutations. These were identified as putatively neutral SNPs. Of these 1,595 potential loci,

1,151 were suitable for MIP-probe design. 76 loci were shared between phylogeographically informative and putatively neutral loci.

MIP capture, amplification, and sequencing

Oligonucleotides described in Supplementary Data 3 were synthesized as 200 nm ultramers (Integrated DNA Technologies, USA) with equimolar hand-mix option for random bases. Upon receipt, these were pooled at equal concentrations to create the MIP panel. MIPs were 5' phosphorylated using 1 µl (10 units) T4 Polynucleotide Kinase (NEB, catalog # M0201) for every nanomole of probe, in 1X T4 DNA ligase buffer (NEB, catalog # B0202S) in a maximum of 50 µl reaction (bigger reactions were split). Phosphorylation reactions were incubated in a thermocycler at 37°C for 45 min followed by heat inactivation at 65°C for 20 min. Probes were aliquoted and kept at -20°C. Probes were diluted 1:8 in TE buffer to bring them to 1 µM working solution. Antimalarial drug resistance and genome wide SNP panels were used in separate reactions.

Capture reactions were carried out as follows. 10 µl capture reactions for each sample and MIP panel containing Ampligase Buffer (1X), Phusion DNA polymerase (0.0008 units/µl), Ampligase (0.04 units/µl), pooled MIPs (40 nM, each), dNTP (4 µM), template DNA (5µl) were incubated in a preheated thermocycler with the following steps 95°C (10 min), 60°C (1 hr), 4°C hold. Next, 2 µl of exonuclease mix containing 1X Ampligase buffer, 10 units Exonuclease I and 50 units Exonuclease III were added to reactions following MIP captures. Reactions were performed in a thermocycler with the following steps: 37°C (1 hr), 95°C (2 min), 4°C hold.

The entire capture reaction (12 µl) was amplified in a 25 µl PCR reaction containing the following components: 1X Phusion Polymerase Buffer, 1X Macromolecular Crowding (MMC) solution, 200 nM dNTP, 0.02 units/µl Phusion DNA polymerase, forward and reverse primers[1], 500 nM each. PCR was performed using a preheated thermocycler with the following steps 98°C 30 s, 22 cycles (98°C 10 s, 63°C 30 s, 68°C 30 s), 68°C 2 min, 4°C hold. 50 ml 5X MMC was prepared by mixing the following components in water and filter-sterilized using 0.2 µ nylon syringe filter: 3.75 g Ficoll 70 (GE Healthcare, catalog # 17-0310-10), 1.25 g Ficoll 400 (Sigma catalog # F2637-5G), 0.125 g Polyvinylpyrrolidone (PVP360, Sigma catalog # PVP360-100g).

Next, library pools were created by combining 5 μl of each PCR reaction in a single tube and cleaned up and concentrated using Ampure XP beads (Beckman Coulter, Catalog #A63881) at 0.8x bead:DNA ratio using manufacturer's protocol. This generally removed the unwanted adapter/primer dimers ~ 200 bp. If dimers remained after bead clean up, the eluted DNA was loaded on a 1.5% agarose gel and the relevant band was extracted from the gel using Monarch DNA extraction kit (NEB, catalog # T1020S). Drug resistance MIP libraries were sequenced on Illumina MiSeq instrument using 250 bp paired end sequencing with dual indexing using MiSeq Reagent Kit v2. Genome-wide MIP libraries were sequenced on Illumina Nextseq 500 instrument using 150 bp paired end sequencing with dual indexing using Nextseq 500/550 Mid-output Kit v2.

MIP data processing and variant calling: Sequencing data was processed using MIPWrangler software [Version 1.1.1-dev, Hathaway, unpublished] in combination with other software. Briefly, sequences were demultiplexed by their dual sample barcode using bcl2fastq software (v2.20.0.422, Illumina). Paired end reads were then stitched together using MIPWrangler and filtered on expected length and on per base quality scores by discarding a sequence if the fraction of quality scores above 30 was less than 70% (Q30 <70%). Quality filtered stitched sequences were then further demultiplexed by target using the extension and ligation arm sequences to produce a file for each target for each sample. Target sequences for each sample were then corrected using their unique molecular identifiers (UMIs). This was done by clustering sequences on their UMIs and then creating a consensus sequence for each specific UMI. This UMI redundancy removes a significant proportion of PCR errors that occur in late cycles, including polymerase stutter and subsequent sequencing errors. UMI corrected sequences were then further clustered within MIPWrangler using an implementation of the qluster algorithm derived from SeekDeep[12] allowing accurate detection of single base differences and indels at levels of 1% or less. We set a minimum relative abundance threshold of 0.5% for a cluster to be included in final analysis. Differences between the observed sequence and the reference sequence for each probe were obtained by pairwise alignment using LastZ software (version 1.04.00)[13] and nucleotide variants and indels from the LastZ output were annotated using Annovar software (version 20180416)[14].

Complexity of Infection: We applied THE REAL McCOIL (v2) categorical method to the SNP genotyped samples to estimate each individual's COI[15], using a 10% minor allele frequency

cutoff for calling a heterozygous locus. We performed five repetitions for each sample, with a burn-in period of 10^4 iterations followed by 10^6 sampling iterations and using standard methodology to confirm convergence between chains[16]. Given the concurrent estimation of population allele frequencies within THE REAL McCOIL, samples were grouped initially within their countries. Default priors were assigned for each parameter, with a maximum observable COI equal to 25 and sequencing measurement error estimated along with COI and allele frequencies. COI estimates were compared between countries using 100,000 repetitions of a non-parametric bootstrap to estimate the 95% confidence interval from the bootstrapped COI density. Additionally, to test for a relationship between COI and transmission intensity, we modelled the exponential relationship between COI and malaria prevalence in the DRC at the cluster level, with a random intercept for each administrative region.

Extended haplotype homozygosity analysis: Alleles at these biallelic SNPs were polarized as ancestral versus derived using P. reichenowi as an outgroup. Briefly, the *P. falciparum* 3D7 assembly was aligned to the PlasmoDB v38 assembly of the *P. reichenowi* CDC strain with nucmer[17] using parameters "-g 500 -c 500 -l 10" as in Otto et al.[18] Only segments with globally unique, one-to-one alignments were retained. The *P. reichenowi* allele was defined as ancestral at all SNPs in these segments; SNPs falling outside these segments were considered to have ambiguous ancestral state and were excluded from analysis. In order to account for linkage between SNPs, we created a recombination map from the pedigrees reported in Miles et al.[19] under the assumption of no unobserved double-crossovers. The genetic positions of SNPs in the MIP panel were interpolated on this map with piecewise-linear interpolation.

We then subsetted to a set of monoclonal samples as identified by THE REAL McCOIL categorical method. Haplotypes were created from the genotype calls, which were the majority within-sample allele frequency. To account for population structure, we analyzed the samples with respect to country with the exception of the DRC, which was split into two groups using K-means clustering weighted by longitude- and latitude-coordinates (**Supplementary Figure 7**). K-means clustering with two groups resulted in an East-West divide that is consistent with previous publications of DRC population substructuring[20]**.** Samples were further subsetted to those without any missing genotype data.

Given that the MIP panel density was not uniform or symmetric by design, we did not perform genome-wide scans for recent positive selection[21,22]. Genome-wide scans for recent positive selection with our MIP panel would have been biased towards sites with higher MIP density and would not have been comparable across the genome. However, given that MIP-site densities are identical between subpopulations at the same genomic region, cross-population interpretations of differing selection pressures were still considered valid. As a result, we calculated the log-ratio of the integrated EHH for the derived allele, hereafter called the $XP\text{-}EHH_D$, to differentiate recent positive selection between subpopulations[23]. We focused on sites that were identified in this study as putative drug-resistance loci and had a prevalence of at least 10% in the DRC. The resulting $XP\text{-}EHH_D$ were then standardized and a one-sided p-value was calculated for each site assuming a Gaussian cumulative distribution[24,25]. We did not perform multiple comparison corrections when evaluating the XP-EHH associated p-values.

Suppplementary Note 2
Structured Discrete-Time Wright Fisher Model for *Plasmodium falciparum* Genetics

Supplementary text accompanying the main paper *The Impact of Antimalarial Resistance on the Genetic Structure of Plasmodium falciparum in the DRC*

Robert Verity, Ozkan Aydemir, Nicholas F. Brazeau, Oliver J. Watson, Nicholas J. Hathaway, Melchior Kashamuka Mwandagalirwa, Patrick W. Marsh, Kyaw Thwai, Travis Fulton, Madeline Denton, Andrew P. Morgan, Jonathan B. Parr, Patrick K. Tumwebaze, Melissa Conrad, Philip J. Rosenthal, Deus S. Ishengoma, Jeremiah Ngondi, Julie Gutman, Modest Mulenga, Douglas E. Norris, William J. Moss, Benedicta A Mensah, James L Myers-Hansen, Anita Ghansah, Antoinette K Tshefu, Azra C. Ghani, Steven R. Meshnick, Jeffrey A. Bailey, Jonathan J. Juliano

March 12, 2020

1 Introduction

Here we describe a simple model that can be used to explore various aspects of *P. falciparum* genetics. This model can be used to simulate populations of human hosts carrying potentially multiple distinct *P. falciparum* haplotypes, taking into account both super-infection and co-transmission. The "true" simulated haplotypes can then be passed through an observation model to

create simulated genotypes that are somewhat representative of real data. The advantage of using simulated data in this way is that the true relatedness between samples is known, and hence methods of estimating relatedness can be compared against a known ground truth.

We start by describing the underlying model from a traditional perspective, emphasising the link to models of population structure. We then argue that this model can be reinterpreted for the purposes of modeling *P. falciparum*. Next we describe recombination and identity by descent within this framework, and finally we describe the observation model that is applied to simulated haplotypes to arrive at more realistic genotypes.

2 The Underlying Structured Model

The model description here is phrased in terms of traditional population genetics, for example describing migration of individuals between demes. However, bear in mind that in the next section the meaning of these terms will be recast in terms of *P. falciparum*.

We assume a discrete-time model with time indexed by $t \in Z_{\geq 0}$ generations. Each generation consists of N demes, and each deme contains one or more distinct individuals. Let the number of individuals in deme $i \in 1:N$ at time t be written $n_{t,i}$. We initialise the model at time $t = 0$ with $n_{0,i}$ drawn from a zero-truncated Poisson distribution with rate λ:

$$\Pr(n_{0,i}) = \frac{\lambda^{n_{0,i}} e^{-\lambda}}{n_{0,i}!(1 - e^{-\lambda})}. \tag{1}$$

At time t, individuals within a deme come together in pairs, chosen at random, and mate to form a single offspring per pair through recombination. The number of times this occurs per deme is denoted K. Offspring then undergo migration. Each offspring stays in its current deme with probability $(1-m)$, or migrates with probability m, in which case it has an equal chance of migrating to each of the N demes. Note that offspring can "migrate" to their current deme, meaning the probability of staying put is actually $(1 - m + m/N)$.

After migration is complete, offspring within each deme are culled down at random to produce the new population size. The number of surviving offspring within each deme, $n_{t,i}$, is drawn from a zero-truncated Poisson distribution with rate λ, exactly the same as in the first generation. We take the limit of this model as $K \to \infty$, thereby ensuring there are always sufficient offspring to be culled down to any given population size.

Although the description above specifies the true forwards-in-time model, it is usually more convenient to think about this process in terms of how generation $t \in Z_{>0}$ relates to generation $t-1$. At time t we can immediately draw the population size $n_{t,i}$ from a zero-truncated Poisson distribution with rate λ. Then, for each individual $j \in 1 : n_{t,i}$ we can draw the parental deme (denoted d) from the following categorical distribution:

$$\Pr(d) = \begin{cases} 1 - m + \frac{m}{N}, & \text{if } d = i \ , \\ \frac{m}{N}, & \text{if } d \neq i \ , \end{cases} \quad \text{for } d \in 1 : N. \tag{2}$$

Finally we can draw the two parents of individual j from deme d with equal probability $1/n_{t-1,d}$. Let these parents be indexed by a_1 and $a_2 \in 1 : n_{t-1,d}$. We repeat this process for every deme, resulting in a new population at time t. Crucially, this process is mathematically equivalent to the forwards-in-time process described above in $\lim_{K\to\infty}$, but is both simpler and more computationally efficient because we never deal with the offspring that are eventually culled.

This model has a standard description in classical population genetics as a structured Wright-Fisher model. Such models have a long history of being used to describe organisms that are split into partially isolated subpopulations [26], where the parameter m is a migration rate that allows us to transition from perfect isolation when $m = 0$ to panmixia when $m = 1$. The only idiosyncrasies of the model above are that 1) we model fluctuating population sizes per deme using the zero-truncated Poisson distribution, and 2) we assume migration occurs before culling, while some models assume it occurs after culling.

3 Recasting the Model for *P. falciparum*

Human hosts can be thought of as populations of malaria parasites, hence we can recast the model above by treating hosts as demes. In this context, N becomes the number of human hosts and $n_{t,i}$ becomes the complexity of infection (COI) of host i at time t. λ becomes the mean COI over the population, which will typically be small (usually ≤ 5).

We must also rethink the continuity of demes over time. In the traditional perspective, deme i at time t represents the same fundamental unit as deme i at time $t+1$, but when applying to $P. falciparum$ we assume that hosts are independent between generations, meaning these refer to completely different hosts. The idea of "migration" therefore needs to be reinterpreted,

as recombinant products that pass from host i at time t to host i at time $t+1$ are not "staying put" as they were in the original formulation. The parameter m is now better understood as a tuning parameter that controls the extent to which hosts are infected by multiple haplotypes transmitted from the same source host (when m is close to 0), vs. hosts being infected by haplotypes contributed from a large number of source hosts (when m is close to 1). The former roughly equates to co-transmission of haplotypes, while the latter equates to super-infection, and the tuning parameter m allows us to vary the balance of these effects. The probability of super-infection is known to increase at high transmission, therefore we can roughly model high transmission settings by using large values of m and λ, vs. low transmission settings with small values of m and λ.

4 Ancestry and Recombination

Our aim is to simulate blocks of identity by descent (IBD) between haplotypes within the model framework described above. Haplotypes are modeled as vectors of length L, corresponding to genomic positions g_l for $l \in 1 : L$. The jth haplotype within host i at time t will be written $\mathbf{x}_{t,i,j}$ for $j = 1 : n_{t,i}$ and the individual elements of this vector will be indexed using $x_{t,i,j,l}$ for $l \in 1 : L$.

IBD is always defined relative to some starting population, therefore at time $t = 0$ we give each haplotype a unique value:

$$x_{0,i,j,l} = j + \sum_{\substack{k=1 \\ k \neq i}}^{i} n_{0,k} \ , \quad \forall l \ . \tag{3}$$

For the recombination model, we assume a constant hazard ρ over the continuous interval $[0, L]$, resulting in a total number of recombination breakpoints $n_b \sim \text{Poisson}(L\rho)$. We then draw the genomic positions of all n_b breakpoints independently from a Uniform$(0, L)$ distribution. Let $b_1, ..., b_{n_b}$ be the positions of these breakpoints, already sorted into ascending order. We use these positions to create a set S of mutually exclusive intervals such that:

$$S = \{[0, b_1), [b_1, b_2), ..., [b_{n_b-1}, b_{n_b}), [b_{n_b}, L)\}. \tag{4}$$

We assume that paternal vs. maternal ancestry alternates over subsequent intervals. Let each interval S_i have a corresponding ancestry A_i. Recalling that we defined a_1 and a_2 as the ancestors of haplotype j in deme d of the previous generation, we can define:

$$A_i = \begin{cases} a_1, & \text{if } i \text{ is odd }, \\ a_2, & \text{if } i \text{ is even }. \end{cases} \tag{5}$$

Finally, we relate the genomic positions g_l back to the intervals in S. Let c_l for $l \in 1 : L$ index the interval in which position g_l falls, i.e. position g_l falls within interval S_{c_l}. At this stage we have everything needed to relate haplotypes at time t back to haplotyes at time $t-1$. This can be done through the expression:

$$x_{t,i,j,l} = x_{t-1,d,A_{c_l},l} \ , \quad \forall l \ . \tag{6}$$

This simply states that the value of a haplotype at a given locus at time t is equal to the value of its ancestor at time $t-1$. We evaluate this expression over every l, j, i and t, and in doing so we simulate the buildup of shared ancestry forwards in time.

5 Identity by Descent

IBD between hosts can be defined in terms of shared ancestry in the haplotypes contained within those hosts. Let $I_{t,u,v}$ be a vector of length L giving the IBD status at each locus at time t between hosts u and v. It will be indexed with elements $I_{t,u,v,l}$ for $l \in 1 : L$, taking values 0 for non-IBD vs. 1 for IBD. Here we say that two hosts are IBD if *any* pair of haplotypes between these hosts share common ancestry. Hence, we can say:

$$I_{t,u,v,l} = \begin{cases} 1, & \text{if } \exists \left\{ j_1, j_2 : x_{t,u,j_1,l} = x_{t,v,j_2,l} \right\} , \\ 0, & \text{otherwise }. \end{cases} \tag{7}$$

The total IBD proportion between samples u and v at time t is the the mean over this vector:

$$\text{IBD}^t_{u,v} = \frac{1}{L} \sum_{l=1}^{L} I_{t,u,v,l} \ . \tag{8}$$

Note that we have not considered mutation in this model, hence IBD between all samples will build up gradually over time until all samples are IBD from a single common ancestor. This is unrealistic over long timescales, where intervening mutations will limit IBD, causing it to tend to an equilibrium state < 1. For this reason we only apply this model over a small number of generations. Our justification is that we are only interested in samples that share high relatedness relative to the background, and that can

be identified with some confidence. IBD blocks in the distant past are likely to be very small, perhaps spanning just a single genotyped locus, and so will be indistinguishable from background identity by state. Even if we could identify these regions, relatedness at this level gives us little useful information about the recent transmission history of the population. For this reason, we use the arbitrary cutoff of $t = 10$ generations when simulating IBD (see section 7).

6 Observation Model

True IBD as defined above (i.e. $\text{IBD}^t_{u,v}$) takes into account relationships between all pairs of haplotypes within samples. However, the maximum likelihood estimator described in the main text assumes monoclonal samples. For polyclonal samples this forces us to discard samples, or to coerce samples to monoclonal, for example by calling the major allele at every locus. We can test the impact this has on IBD estimates by performing a similar procedure on simulated data prior to evaluating the maximum likelihood estimator. This requires an observation model that takes us from the complete set of phased haplotypes per sample, to a single genotype per sample consisting of within-sample allele frequencies of the reference vs. alternate allele.

First, we assume that all alleles are biallelic, and are drawn from the population allele frequencies p_l for each locus $l \in 1 : L$ (note this is the frequency of the reference allele). We can convert our ancestry vectors $\mathbf{x}_{t,i,j}$ directly to observed alleles by drawing from a finite alleles model for each unique ancestor. Let $\mathbf{w}_l$ be a vector of alleles, which will be indexed via $\mathbf{w}_l[k]$. The length of $\mathbf{w}_l$ is $\sum_{i=1}^{N} n_{0,i}$, as this covers all possible ancestral values at this locus (see (3)). The values $\mathbf{w}_l[k]$ are modeled as independent Bernoulli draws, each with probability p_l. Next we define a vector $\mathbf{y}_{t,i,j}$, which is analogous to $\mathbf{x}_{t,i,j}$ but will contain biallelic values rather than ancestry. We define:

$$y_{t,i,j,l} = \mathbf{w}_l[x_{t,i,j,l}] \ , \quad \forall t, i, j, l \ , \tag{9}$$

in other words, the biallelic haplotypes $\mathbf{y}_{t,i,j}$ are obtained by passing the underlying ancestry $\mathbf{x}_{t,i,j}$ through the finite alleles vector $\mathbf{w}_l$. Any genetic elements that share common ancestry will also share the same allele, but elements that have different ancestry may also share the same allele by chance (i.e. they will be identical by state (IBS)).

Next, we consider the strain frequencies of each haplotype within a sample. Sample i contains $n_{t,i}$ haplotypes, and so we create a corresponding

vector of strain frequencies, $f_{t,i,j}$ for $j \in 1 : n_{t,i}$. These frequencies are drawn from a symmetric Dirichlet distribution with concentration parameter θ:

$$\Pr(\mathbf{f}_{t,i} \mid \theta) = \frac{\Gamma(n_{t,i}\theta)}{\Gamma(\theta)^{n_{t,i}}} \prod_{j=1}^{n_{t,i}} f_{t,i,j}^{\theta-1} \ . \tag{10}$$

The true within-sample allele frequency is obtained by summing over all biallelic haplotypes, weighted by these frequencies. Let $g_{t,i,l}^{\text{true}}$ be the within-sample allele frequency (of the reference allele) of sample i at locus l. This is obtained via:

$$g_{t,i,l}^{\text{true}} = \sum_{j=1}^{n_{t,i}} (y_{t,i,j,l} \times f_{t,i,j}) \ . \tag{11}$$

We assume these true frequencies are perturbed by random errors in sequencing, which occur independently for each locus with probability ε. We define the purturbed within-sample allele frequencies as follows:

$$g_{t,i,l}^{\text{error}} = g_{t,i,l}^{\text{true}}(1-\varepsilon) + (1-g_{t,i,l}^{\text{true}})\varepsilon \ . \tag{12}$$

Finally, we draw read counts at each locus. Let $z_{t,i,l}$ be the read count of sample i at locus l. For the case of molecular inversion probes this is the the observed number of unique molecular identifiers of the reference allele. We assume that read counts are drawn independently for each locus from a Beta-Binomial distribution with total coverage d and shape parameters α and β:

$$\Pr(z_{t,i,l} = k|d,\alpha,\beta) = \frac{\Gamma(d+1)}{\Gamma(k+1)\Gamma(d-k+1)} \frac{\Gamma(k+\alpha)\Gamma(d-k+\beta)}{\Gamma(d+\alpha+\beta)} \frac{\Gamma(\alpha+\beta)}{\Gamma(\alpha)\Gamma(\beta)} \ . \tag{13}$$

The parameters α and β are defined from the purturbed allele frequencies as follows:

$$\alpha = g_{t,i,l}^{\text{error}}/\eta \tag{14}$$

$$\beta = (1-g_{t,i,l}^{\text{error}})/\eta \ , \tag{15}$$

where η is an over-dispersion parameter relative to the ordinary Binomial distribution. The final observed within-sample allele frequency can be obtained by dividing the observed read counts by the total read depth, i.e. we define $g_{t,i,l}^{\text{obs}} = z_{t,i,l}/d$. This will be close to the true frequencies $g_{t,i,l}^{\text{true}}$, but will be biased by sequencing errors and will contain random noise due to

sampling. $g^{\text{obs}}_{t,i,l}$ calculated for samples u and v can be passed to the maximum likelihood estimator described in the main text to arrive at an estimate of IBD. We are interested in how this estimate differs from the true value $\text{IBD}^t_{u,v}$.

7 Parameter Values

The following parameter values and ranges were used in simulation in the main text:

t	10
N	10, 50, 100, 500, 1000
$\lambda/(1-e^{-\lambda})$	1.0, 2.0, 3.0
	(mean COI of positive samples)
m	0.0, 0.25, 0.5, 1.0
L	1079, 500, 100, 20
	(full set of 1079 loci and sequential random sub-samples)
ρ	7.4×10^{-7}
	(from [27])
p_l	drawn independently for each l from $\text{Beta}(1.544, 0.620)$
	(shape parameters fitted from true allele frequency distribution)
θ	1.0
ε	0.05
η	0.10

Gene	Mutation	Locus Position	sXP-EHH	Stat. Sig.
dhfr	N51I	8	0.62	F
dhfr	C59R	9	0.10	F
dhfr	S108N	10	0.27	F
mdr1	N86Y	34	-	-
mdr1	Y184F	39	0.31	F
mdr1	D1246Y	47	-	-
crt	M74I	16	-1.66	T
crt	N75E	17	-1.66	T
crt	K76T	19	-1.66	T
crt	I356T	25	-	-
dhps	S436A	37	0.89	F
dhps	G437A	38	0.40	F
dhps	K540E	39	1.20	F
dhps	A581G	40	0.76	F
mdr2	I492V	5	-0.01	F
mdr2	F423Y	6	0.43	F

Supplementary Table 1 - Cross Population Extended Haplotype Homozygosity Statistics Contrasting the Eastern and Western Democratic Republic of the Congo. The cross-population extended haplotype homozygosity (XP-EHH) statistics contrast the Eastern Democratic Republic of the Congo (DRC) versus the Western DRC subpopulations. The standardized XP-EHH (sXP-EHH) statistic is a standardized log ratio of the extended haplotype homozygosity between the two subpopulations, such that a positive value indicates more extended haplotype homozygosity in the Eastern DRC than the Western DRC. The categorical result of the one-side p-value (α: 0.05), without accounting for multiple testing, is indicated for each locus defined as True (T) or False (F) (Stat. Sig- Statistical Significance).

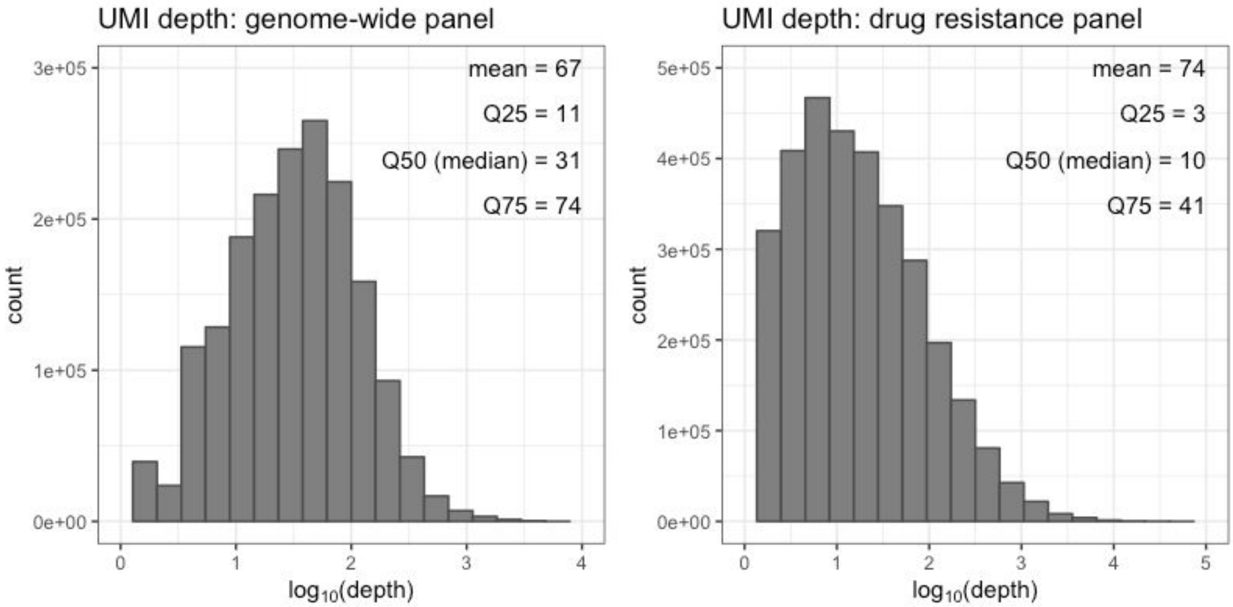

Supplementary Figure 1 - UMI depth distributions. Histograms show the raw distribution of coverage (number of unique UMIs) per locus for the genome-wide and drug resistance MIP panels on a log scale.

(a)

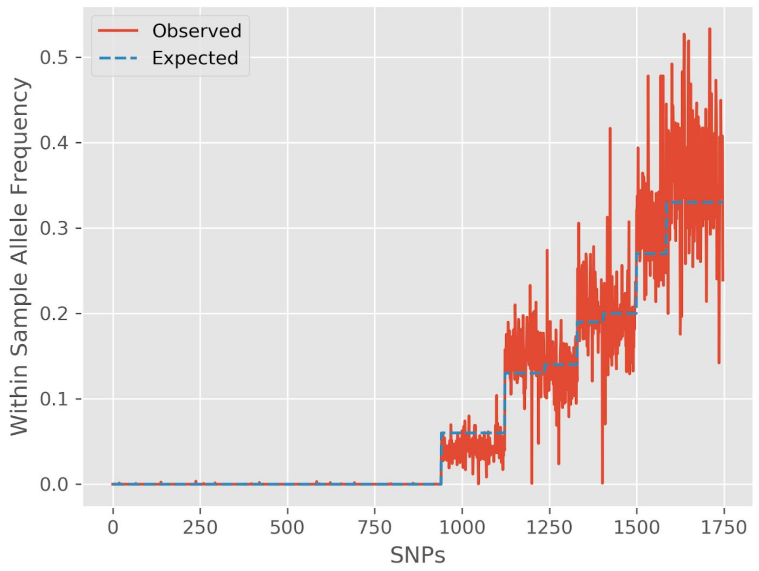

(b)

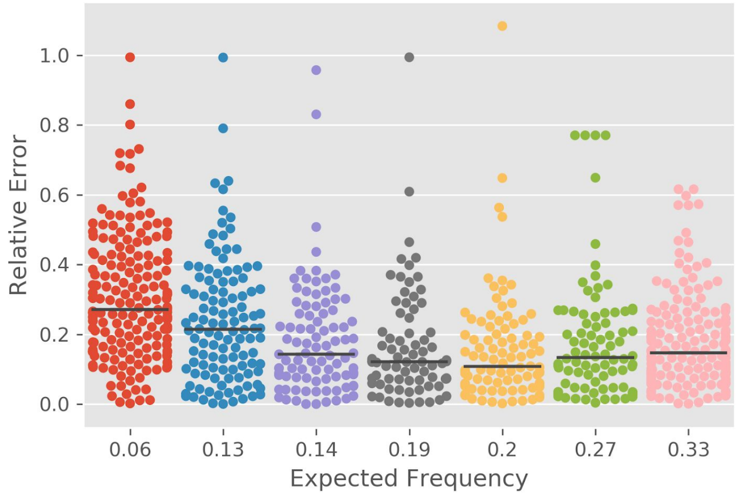

Supplementary Figure 2 - Expected vs. measured allele frequencies in control samples for genome-wide MIPs. A mix of 4 laboratory strains (as described in the methods) were used as controls. **(a)** Each targeted SNP's expected allele frequency (in increasing order) is plotted in blue based on which strains harbor the SNP and what ratio the strain was mixed in the sample. Each SNP's frequency as measured experimentally is plotted in red. Pearson's correlation coefficient between the expected and observed frequencies, calculated for alleles SNPs with nonzero expected frequencies, were 0.925 (R^2=0.856). **(b)** Average relative error was calculated for each SNP using the formula (*experimental mean frequency - expected frequency) / expected frequency*. These values were grouped according to the expected frequencies and all values along with a line indicating the median value were plotted for each group. In total, 114 control reactions were run with experiments.

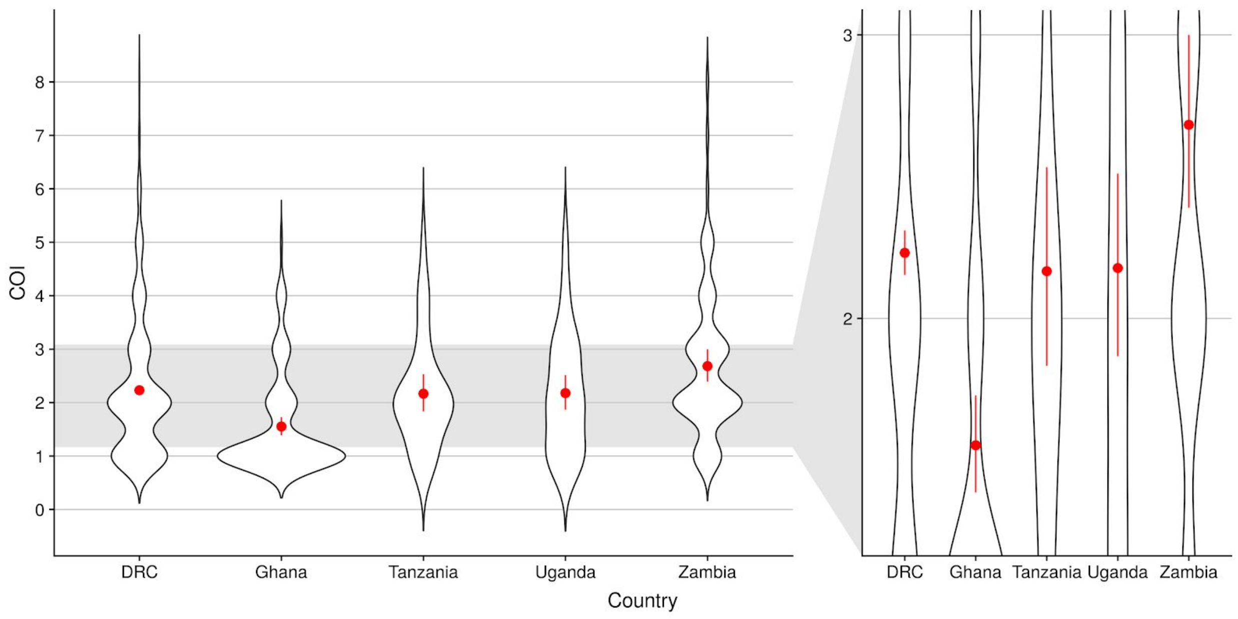

Supplementary Figure 3 - COI distribution per country. Violin plots show the distribution of the estimated COI, which is shown as the median from the posterior distribution estimated using THE REAL McCOIL categorical method. The mean and 95% confidence interval, estimated using a non-parametric bootstrap, are shown in red. Samples collected from Ghana had a significantly lower COI compared to the other countries, and samples collected from Zambia had a significantly higher COI compared to DRC.

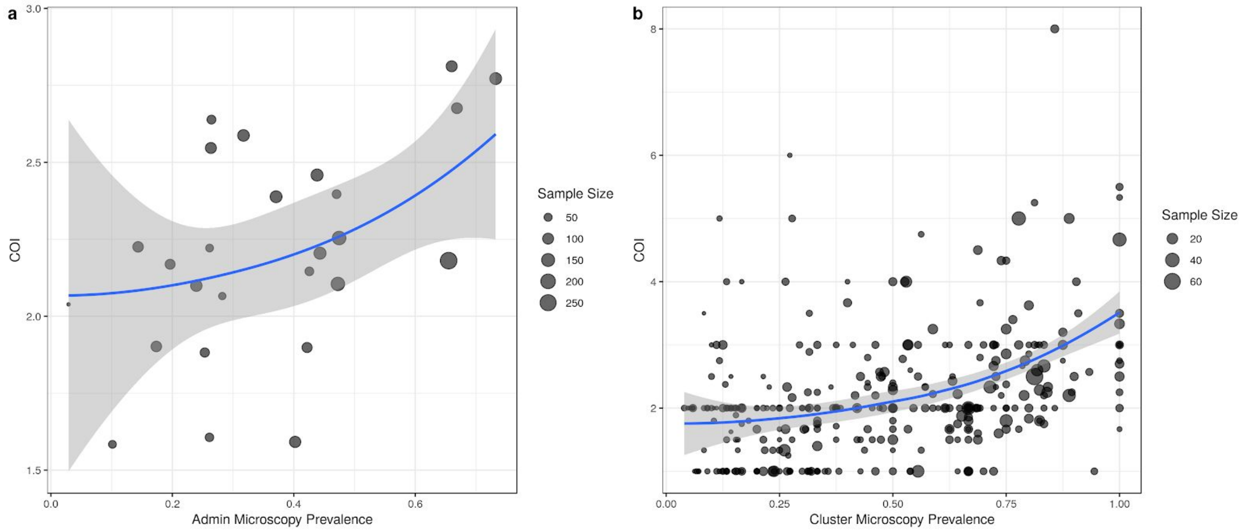

Supplementary Figure 4 - Relationship between COI and prevalence. In **(a)** the relationship between COI and microscopy prevalence at the province level is shown for the samples collected from the DRC. Each point represents the survey-weighted COI estimate and a locally weighted regression is shown in blue with the 95% confidence interval shaded in grey. The same relationship at the cluster level is shown in **(b)** with the size of the points reflecting the survey-weighted sample size.

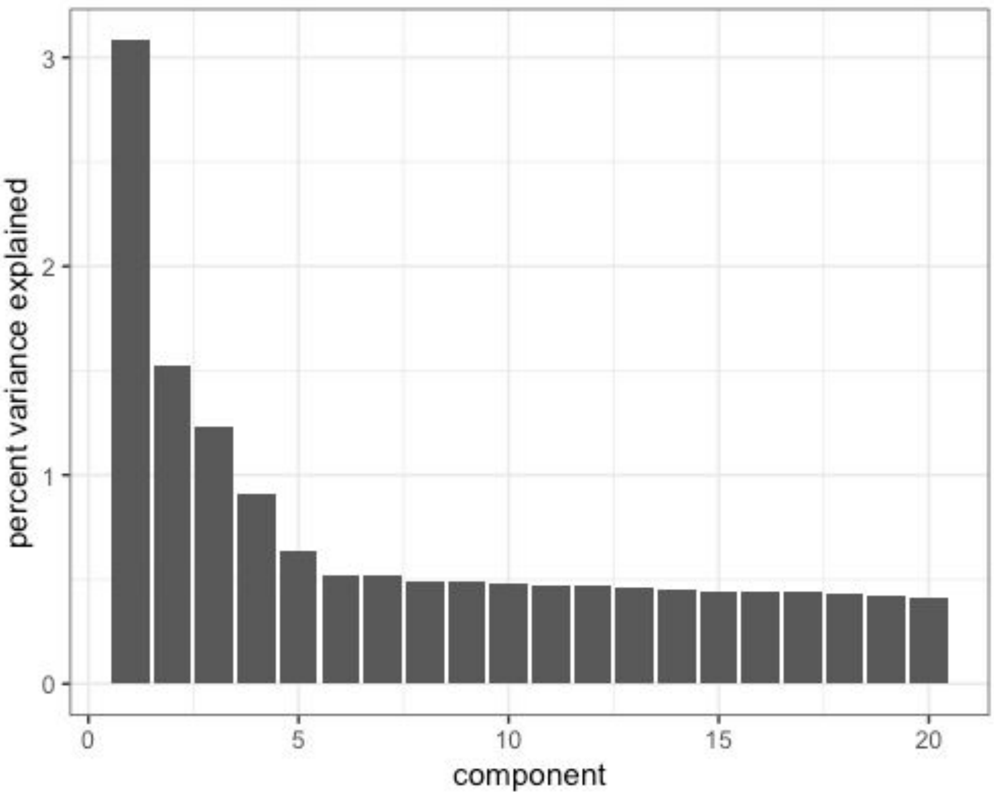

Supplementary Figure 5 - PCA variance explained. The variance explained by the first 20 principal components as a percentage of overall variance.

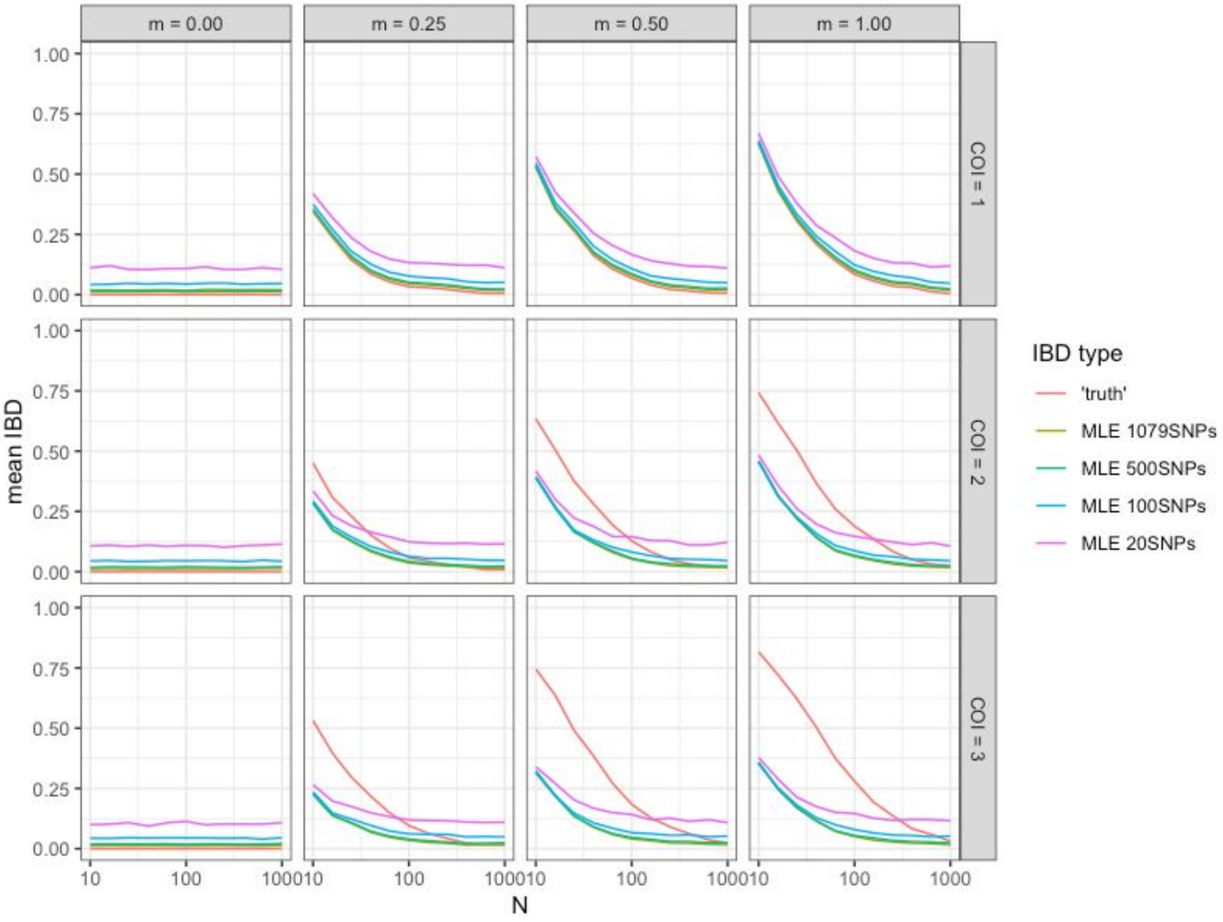

Supplementary Figure 6 - Simulation-based Analysis of IBD. Simulated genetic datasets were generated from a range of values of effective population size (N), mean complexity of infection (COI) and a tuning parameter that relates to super-infection (m) - see **Supplemental Text 2** for full details of the simulation model. The “true” between-sample IBD from the raw (phased) haplotypes is compared against estimated IBD via the maximum likelihood estimator for the full complement of loci, and for smaller subsets.

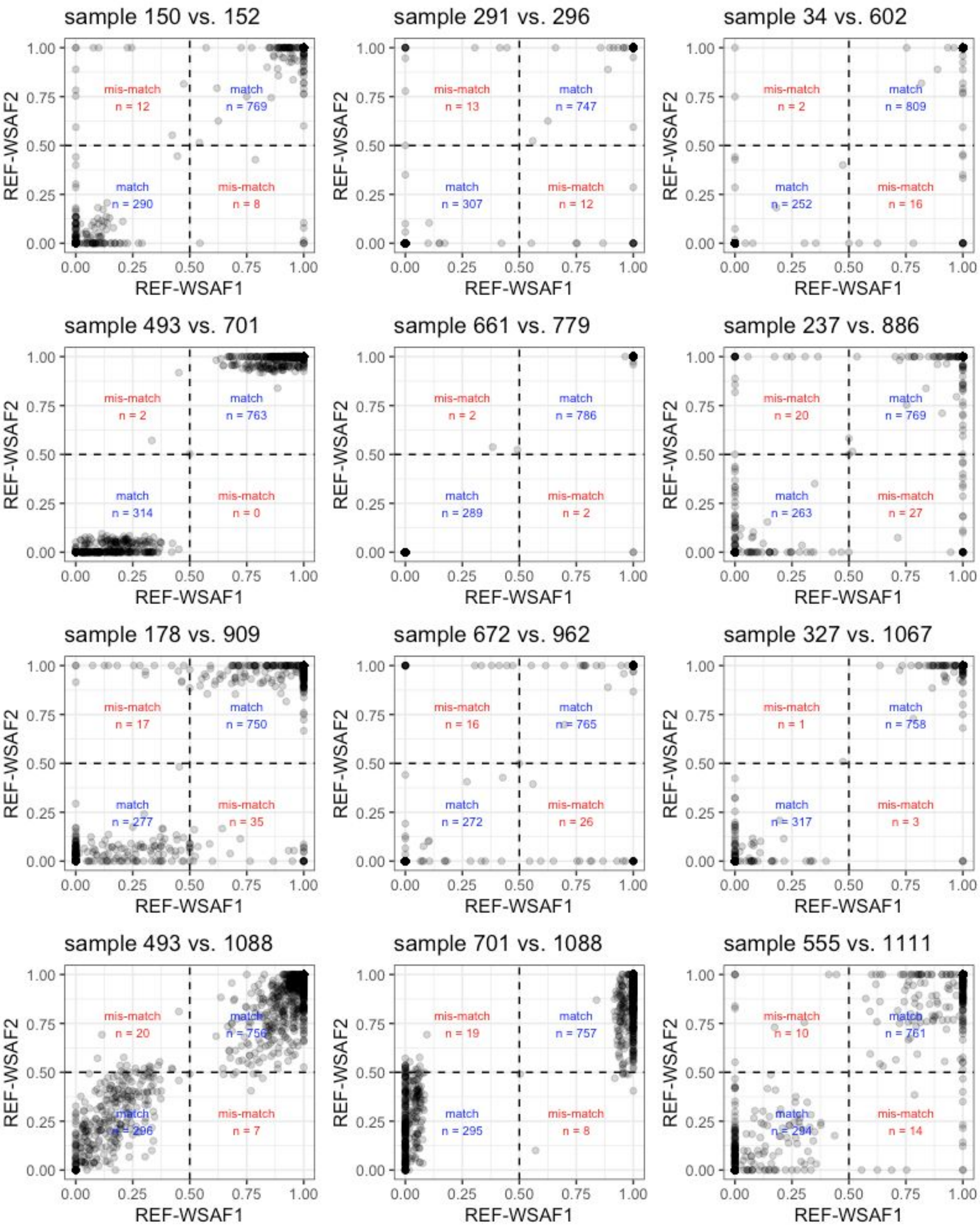

Supplementary Figure 7 - Within-sample allele frequencies of highly related samples. For the 12 sample pairs identified as highly related (IBD>0.9), scatterplots compare the raw within-sample allele frequencies (WSAF) of the referent allele at every locus. Perfectly matching monoclonal genotypes would be represented as a single point in the lower-left and upper-right corners, however, polyclonal infections and sequencing errors cause deviations from this pattern. The number of loci that match or mismatch in terms of occupying the same or different intervals in {[0,0.5), [0.5,1]} is shown.

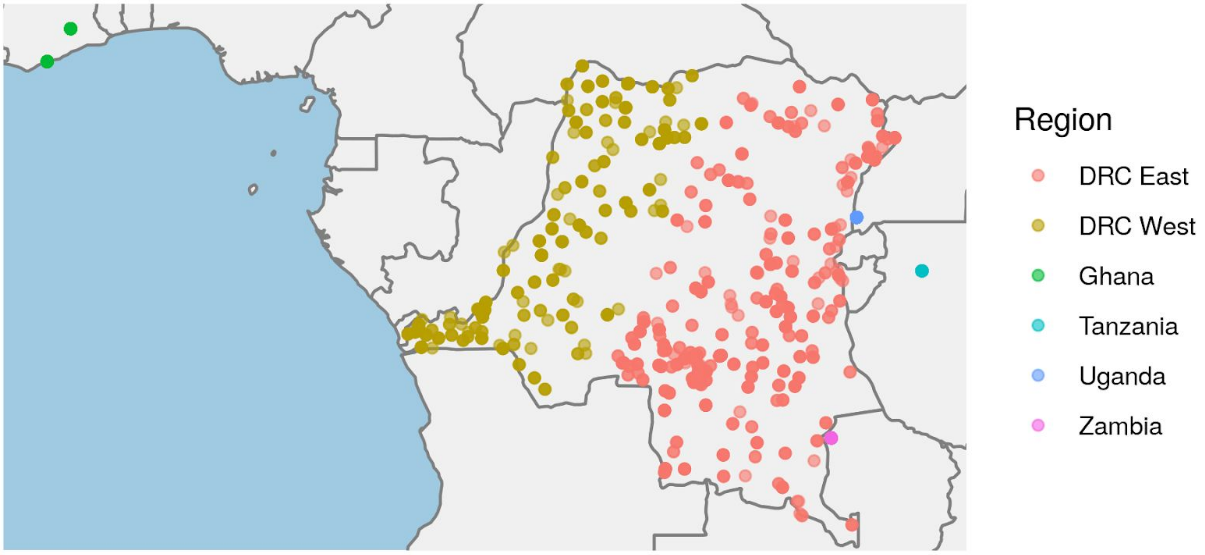

Supplementary Figure 8 - Population K-means clustering. Geographic distribution of samples in each of the two clusters produced by K-means clustering within DRC. Countries outside DRC remain single groups.

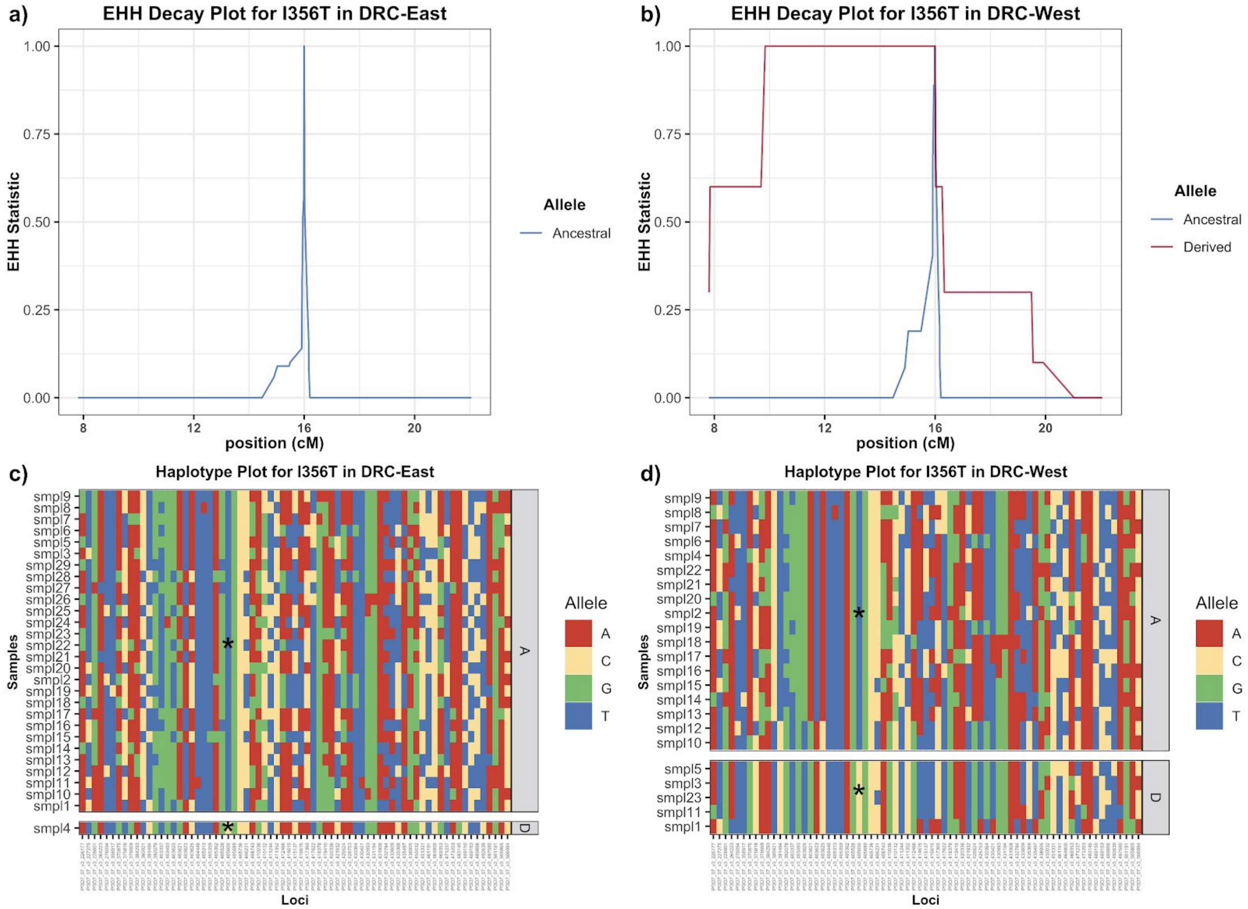

Supplementary Figure 9 - *pfcrt* I356T EHH and haplotype plots among monoclonal infections with no missing genotype data. Panels **(a)** and **(b)** show the extended haplotype homozygosity (EHH) decay plots 200 kilobases upstream and downstream of the I356T core single nucleotide polymorphism (SNP) in centimorgans with respect to the eastern Democratic Republic of the Congo (DRC) and western DRC, respectively. Panels **(c)** and **(d)** display the extended haplotypes with the SNPs colored at each respective loci contributing to the ancestral (A) and derived (D) extended haplotype. The core SNP column is marked with a black asterisks.

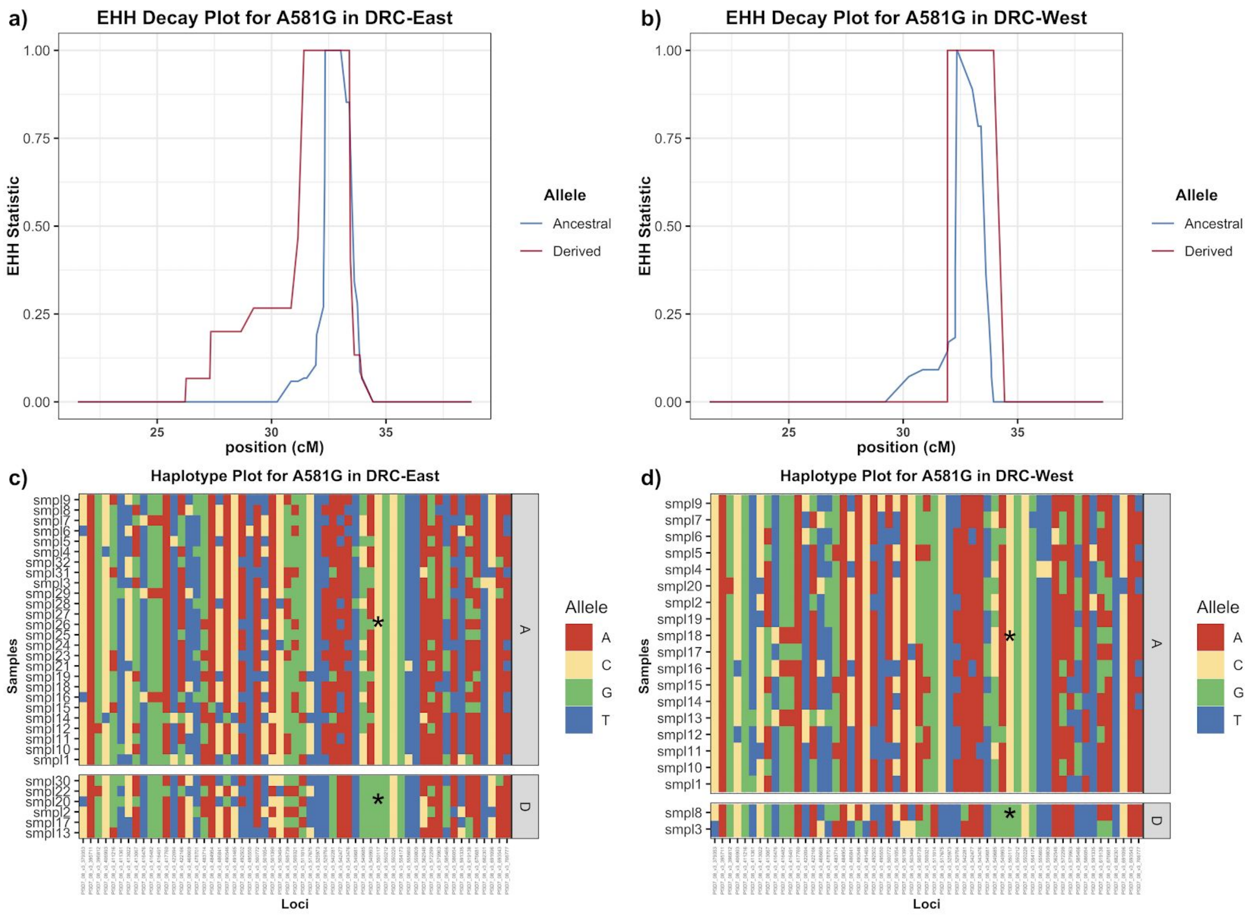

Supplementary Figure 10 - *dhps* A581G EHH and haplotype plots among monoclonal infections with no missing genotype data. Panels (a) and (b) show the extended haplotype homozygosity (EHH) decay curve 200 kilobases upstream and downstream of the A581G core single nucleotide polymorphism (SNP) in centimorgans with respect to the eastern Democratic Republic of the Congo (DRC) and western DRC, respectively. Panels (c) and (d) display the extended haplotypes with the SNPs colored at each respective loci contributing to the ancestral (A) and derived (D) extended haplotype. The core SNP column is marked with a black asterisks.

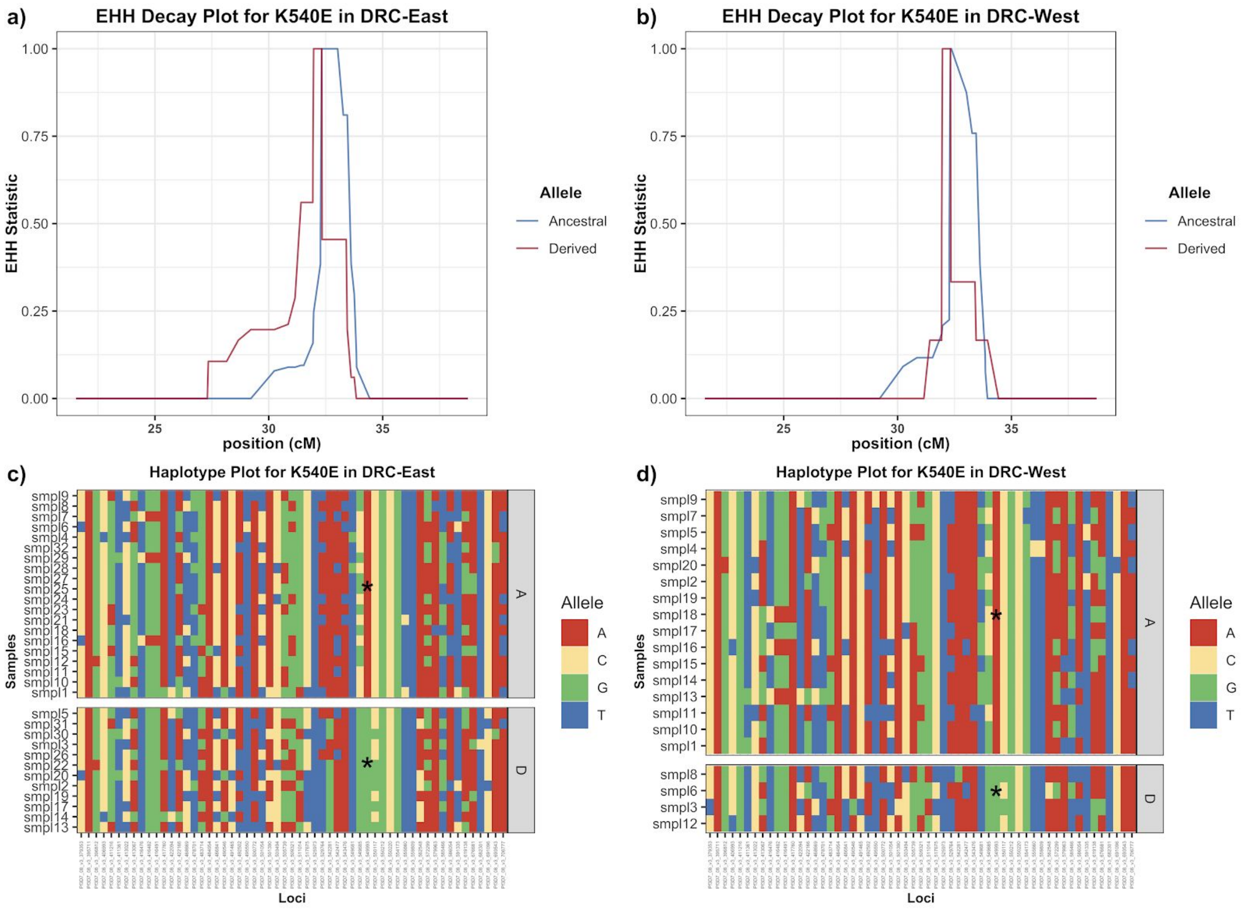

Supplementary Figure 11 - *dhps* K540E EHH and haplotype plots among monoclonal infections with no missing genotype data. Panels (a) and (b) show the extended haplotype homozygosity (EHH) decay curve 200 kilobases upstream and downstream of the K540E core single nucleotide polymorphism (SNP) in centimorgans with respect to the eastern Democratic Republic of the Congo (DRC) and western DRC, respectively. Panels (c) and (d) display the extended haplotypes with the SNPs colored at each respective loci contributing to the ancestral (A) and derived (D) extended haplotype. The core SNP column is marked with a black asterisks.

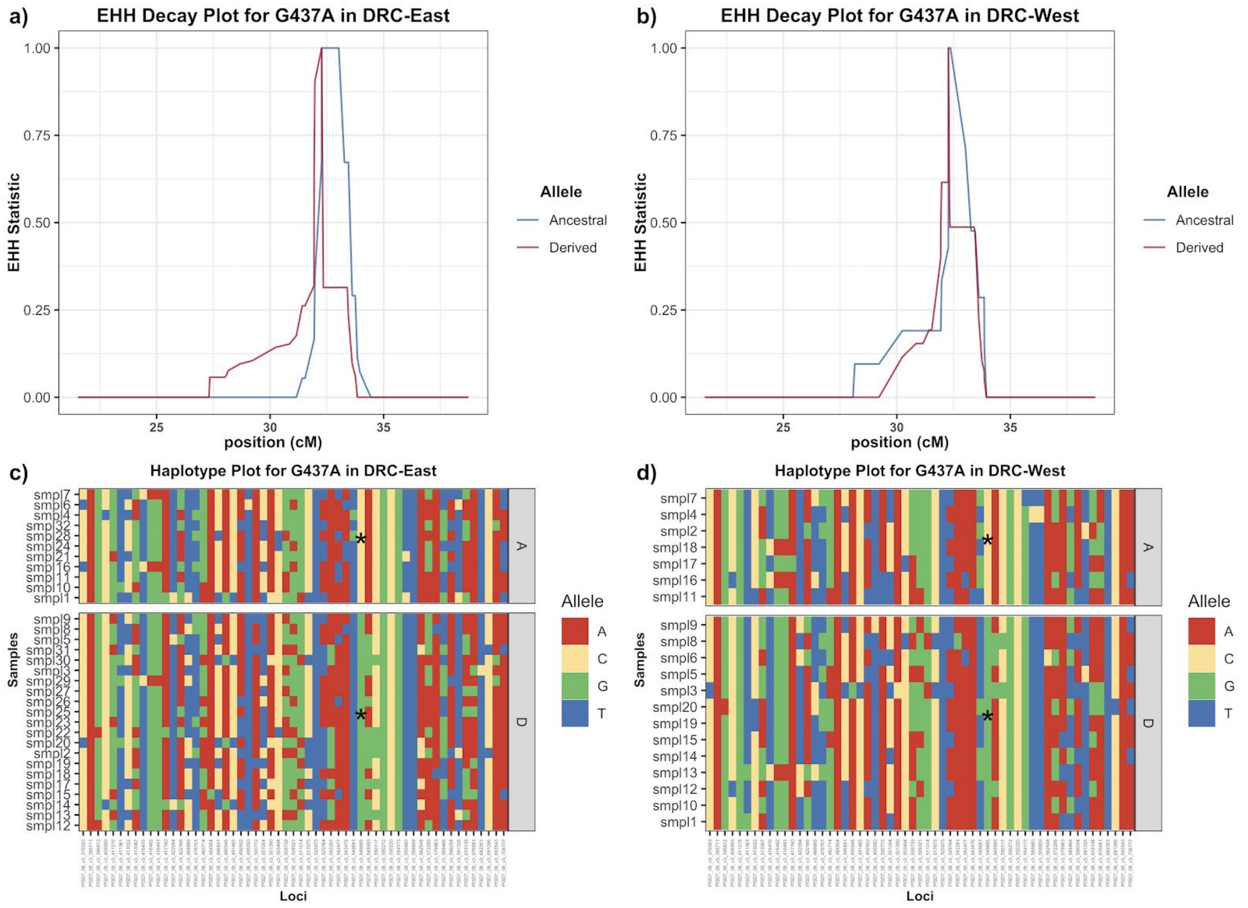

Supplementary Figure 12 - *dhps* G437A EHH and haplotype plots among monoclonal infections with no missing genotype data. Panels (a) and (b) show the extended haplotype homozygosity (EHH) decay curve 200 kilobases upstream and downstream of the G437A core single nucleotide polymorphism (SNP) in centimorgans with respect to the eastern Democratic Republic of the Congo (DRC) and western DRC, respectively. Panels (c) and (d) display the extended haplotypes with the SNPs colored at each respective loci contributing to the ancestral (A) and derived (D) extended haplotype. The core SNP column is marked with a black asterisks.

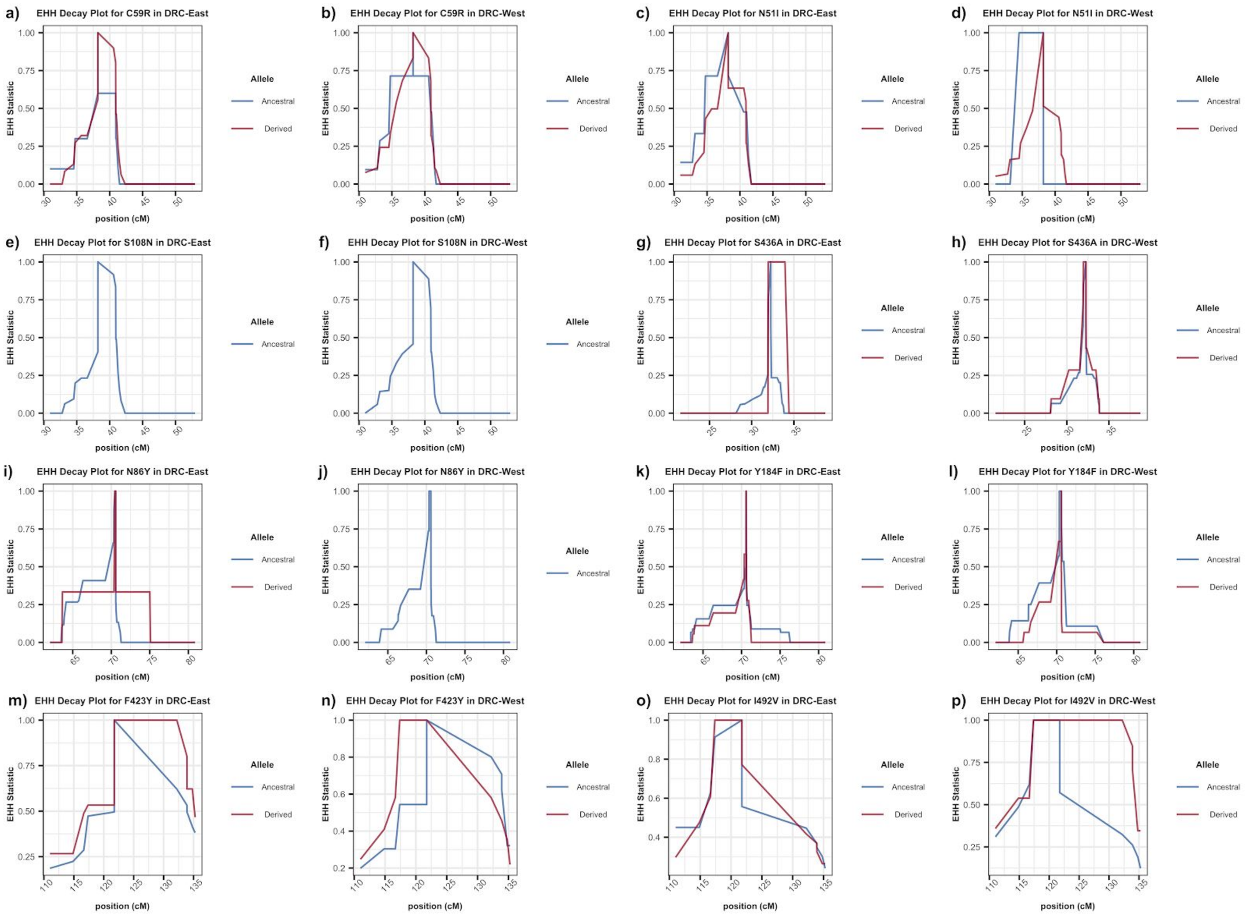

Supplementary Figure 13 - Remaining EHH decay plot for the *mdr1*, *mdr2*, and *dhfr* genes among monoclonal infections with no missing genotype data. Plots (a-j) display the extended haplotype homozygosity (EHH) decay curve 200 kilobases upstream and downstream of the respective core single nucleotide polymorphism. The remaining loci did not appear to be under recent positive selection.

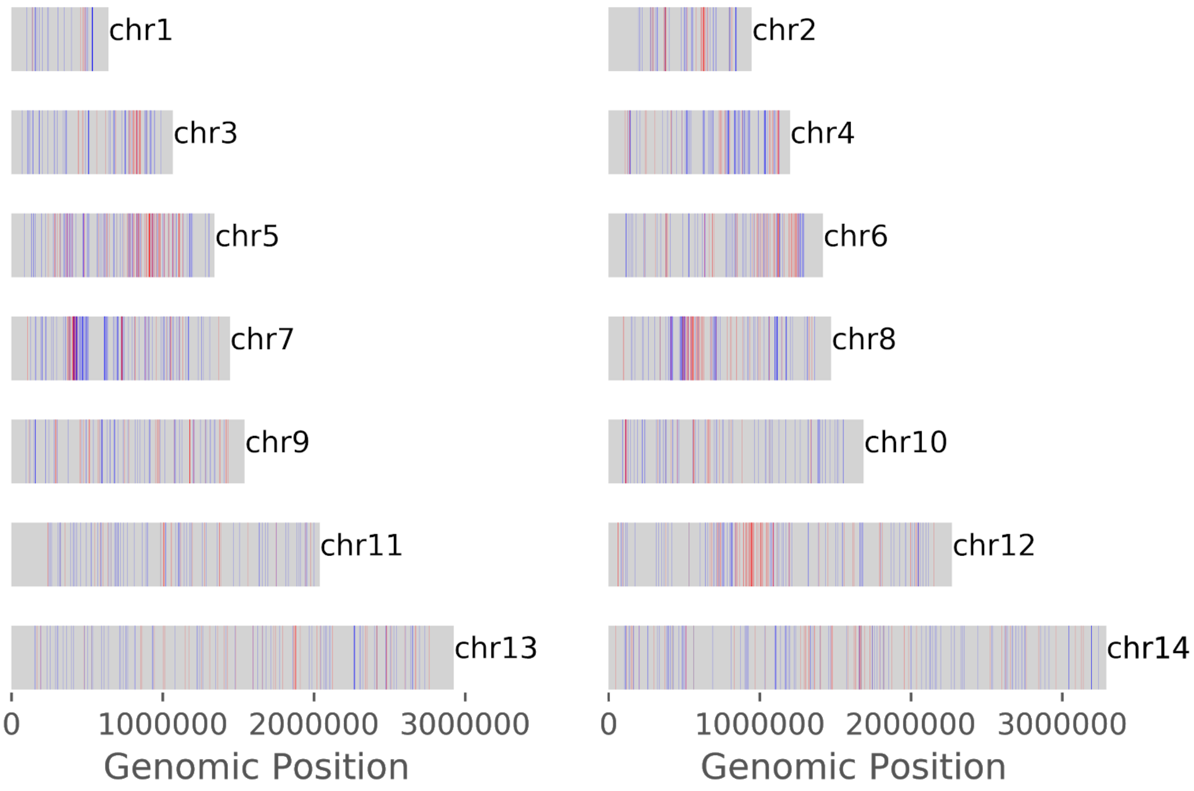

Supplementary Figure 14 - Genomic positions of MIP targets. Genomic locations of geographically informative (red) and putatively neutral (blue) SNP targets are indicated on each chromosome.

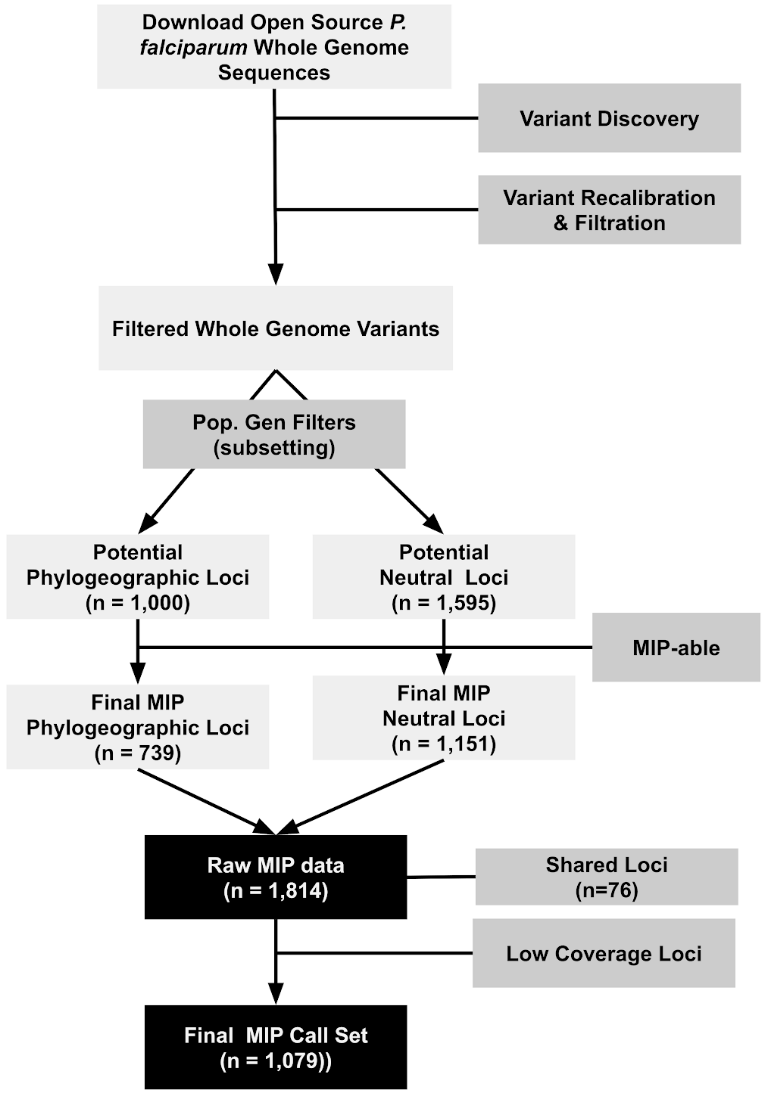

Supplementary Figure 15 - MIP Design and Subsetting Pipeline. Loci were first identified from publicly available data (the Pf3K project) and were subsetted based on population-genetic statistics (light-grey). These loci were then genotyped using molecular inversion probes (MIPs). Loci that had low-coverage from MIP sequence were excluded (black).

Supplementary References

1. Aydemir, O. *et al.* Drug Resistance and Population Structure of Plasmodium falciparum Across the Democratic Republic of Congo using high-throughput Molecular Inversion Probes. *J. Infect. Dis.* (2018) doi:10.1093/infdis/jiy223.
2. MalariaGEN Plasmodium falciparum Community Project. Genomic epidemiology of artemisinin resistant malaria. *Elife* **5**, (2016).
3. The Pf3K Project. *www.malariagen.net/data/pf3k-5* (2016).
4. McKenna, A. *et al.* The Genome Analysis Toolkit: a MapReduce framework for analyzing next-generation DNA sequencing data. *Genome Res.* **20**, 1297–1303 (2010).
5. Van der Auwera, G. A. *et al.* From FastQ data to high confidence variant calls: the Genome Analysis Toolkit best practices pipeline. *Curr. Protoc. Bioinformatics* **43**, 11.10.1–33 (2013).
6. DePristo, M. A. *et al.* A framework for variation discovery and genotyping using next-generation DNA sequencing data. *Nat. Genet.* **43**, 491–498 (2011).
7. Li, H. Aligning sequence reads, clone sequences and assembly contigs with BWA-MEM. *arXiv.org* (2013).
8. Parobek, C. M. *et al.* Selective sweep suggests transcriptional regulation may underlie Plasmodium vivax resilience to malaria control measures in Cambodia. *Proc. Natl. Acad. Sci. U. S. A.* **113**, E8096–E8105 (2016).
9. Li, H. *et al.* The Sequence Alignment/Map format and SAMtools. *Bioinformatics* **25**, 2078–2079 (2009).
10. Picard Tools - By Broad Institute. http://broadinstitute.github.io/picard/.
11. Weir, B. S. & Clark Cockerham, C. Estimating F-Statistics for the Analysis of Population Structure. *Evolution* vol. 38 1358 (1984).
12. Hathaway, N. J., Parobek, C. M., Juliano, J. J. & Bailey, J. A. SeekDeep: single-base resolution de novo clustering for amplicon deep sequencing. *Nucleic Acids Res.* (2017) doi:10.1093/nar/gkx1201.
13. CCGB: Miller Lab, LASTZ. http://www.bx.psu.edu/~rsharris/lastz/.
14. Wang, K., Li, M. & Hakonarson, H. ANNOVAR: functional annotation of genetic variants from high-throughput sequencing data. *Nucleic Acids Res.* **38**, e164 (2010).
15. Chang, H.-H. *et al.* THE REAL McCOIL: A method for the concurrent estimation of the complexity of infection and SNP allele frequency for malaria parasites. *PLoS Comput. Biol.* **13**, e1005348 (2017).
16. Gelman, A. & Rubin, D. B. Markov chain Monte Carlo methods in biostatistics. *Stat. Methods Med. Res.* **5**, 339–355 (1996).
17. Kurtz, S. *et al.* Versatile and open software for comparing large genomes. *Genome Biol.* **5**, R12 (2004).
18. Otto, T. D. *et al.* Genome sequencing of chimpanzee malaria parasites reveals possible pathways of adaptation to human hosts. *Nat. Commun.* **5**, 4754 (2014).
19. Miles, A. *et al.* Indels, structural variation, and recombination drive genomic diversity in Plasmodium falciparum. *Genome Research* vol. 26 1288–1299 (2016).
20. Taylor, S. M. *et al.* Plasmodium falciparum sulfadoxine resistance is geographically and genetically clustered within the DR Congo. *Sci. Rep.* **3**, 1165 (2013).
21. Sabeti, P. C. *et al.* Detecting recent positive selection in the human genome from haplotype structure. *Nature* **419**, 832–837 (2002).
22. Voight, B. F., Kudaravalli, S., Wen, X. & Pritchard, J. K. A map of recent positive selection

in the human genome. *PLoS Biol.* **4**, e72 (2006).
23. Sabeti, P. C. *et al.* Genome-wide detection and characterization of positive selection in human populations. *Nature* vol. 449 913–918 (2007).
24. Gautier, M. & Naves, M. Footprints of selection in the ancestral admixture of a New World Creole cattle breed. *Mol. Ecol.* **20**, 3128–3143 (2011).
25. Gautier, M., Klassmann, A. & Vitalis, R. rehh 2.0: a reimplementation of the R package rehh to detect positive selection from haplotype structure. *Mol. Ecol. Resour.* **17**, 78–90 (2017).
26. Wakeley, John. "Coalescent theory." *Roberts & Company* (2009).
27. Taylor, Aimee R., et al. "Estimating relatedness between malaria parasites." *Genetics* 212, 1337-1351 (2019).
