# Supplementary material for: The impact of antimalarial resistance on the genetic structure of Plasmodium falciparum in the DRC
Source: Nat Commun. 2020 Apr 30;11:2107. doi: 10.1038/s41467-020-15779-8 (PMC7192906; doi:10.1038/s41467-020-15779-8)
Supplement: Supplementary file 3 — Reporting Summary [file 41467_2020_15779_MOESM3_ESM.pdf]

nature research

Corresponding author(s): Juliano, Jonathan James and Verity, Robert

Last updated by author(s): Mar 13, 2020

Reporting Summary

Nature Research wishes to improve the reproducibility of the work that we publish. This form provides structure for consistency and transparency in reporting. For further information on Nature Research policies, see Authors & Referees and the Editorial Policy Checklist .

Statistics

For all statistical analyses, confirm that the following items are present in the figure legend, table legend, main text, or Methods section.

n/a	Confirmed	
☐	☒	The exact sample size (*n*) for each experimental group/condition, given as a discrete number and unit of measurement
☐	☒	A statement on whether measurements were taken from distinct samples or whether the same sample was measured repeatedly
☐	☒	The statistical test(s) used AND whether they are one- or two-sided<br>*Only common tests should be described solely by name; describe more complex techniques in the Methods section.*
☐	☒	A description of all covariates tested
☒	☐	A description of any assumptions or corrections, such as tests of normality and adjustment for multiple comparisons
☐	☒	A full description of the statistical parameters including central tendency (e.g. means) or other basic estimates (e.g. regression coefficient) AND variation (e.g. standard deviation) or associated estimates of uncertainty (e.g. confidence intervals)
☐	☒	For null hypothesis testing, the test statistic (e.g. *F*, *t*, *r*) with confidence intervals, effect sizes, degrees of freedom and *P* value noted<br>*Give P values as exact values whenever suitable.*
☒	☐	For Bayesian analysis, information on the choice of priors and Markov chain Monte Carlo settings
☐	☒	For hierarchical and complex designs, identification of the appropriate level for tests and full reporting of outcomes
☐	☒	Estimates of effect sizes (e.g. Cohen's *d*, Pearson's *r*), indicating how they were calculated

Our web collection on statistics for biologists contains articles on many of the points above.

Software and code

Policy information about availability of computer code

Data collection	Not applicable.
Data analysis	1. General tools for MIP variant calling and filtering are available at https://github.com/bailey-lab/MIPTools (v.0.19.12.13) and https://github.com/Mrc-ide/mipanalyzer. (v1.0.0)<br>- MIP Wrangler (v1.1.1-dev)<br>2. Code and data is available for each figure at https://github.com/bobverity/antimalarial_resistance_DRC.<br>3. Genome Analysis To olkit (GATK) (ver 3.6)<br>4. REAL McCOIL (v2) using McCOILR (v1.3.0)<br>5. prcomp (R version 3.5.1)<br>6. PrevMap (version 1.4.2)<br>7. UpSetR (version 1.3.3)<br>8. rehh (v 2.0.4)<br>9. Picard Tools Mark Duplicates (v 2.2.4)<br>10. MergeSamFiles (version 2.2.4)<br>11. GATK IndelRealigner (version 3.6)<br>12. GATK BaseRecalibrator (version 3.6)<br>13. GATK Haplotypecaller (version 3.6)<br>14. GATK GenotypeGVCFs tool (version 3.6)<br>15. GATK VariantRecalibrator (version 3.6)<br>16. GATK ApplyRecalibration (version 3.6)<br>17. GATK CallableLoci (version 3.6)<br>18. GATK CoveredByNSamplesSites (version 3.4.46)<br>19. snpEFF (version 4.3s)

20. bcl2fastq (v2.20.0.422)
21. LastZ (version 1.04.00)
22. nucmer as a part of MUMMER (version 3.0)
23. SeekDeep
24. Annovar (version 20180416)

For manuscripts utilizing custom algorithms or software that are central to the research but not yet described in published literature, software must be made available to editors/reviewers. We strongly encourage code deposition in a community repository (e.g. GitHub). See the Nature Research guidelines for submitting code & software for further information.

Data

Policy information about availability of data

All manuscripts must include a data availability statement. This statement should provide the following information, where applicable:

- Accession codes, unique identifiers, or web links for publicly available datasets
- A list of figures that have associated raw data
- A description of any restrictions on data availability

1. Sequencing reads have been deposited into the NCBI SRA (Accession numbers: PRJNA454490, PRJNA545345 and PRJNA545347).
2. DHS data for the 2013 DRC DHS is available here: https://dhsprogram.com/what-we-do/survey/survey-display-421.cfm (This includes clinical and GPS information and is available upon request fro the DHS program.)

Field-specific reporting

Please select the one below that is the best fit for your research. If you are not sure, read the appropriate sections before making your selection.

☐ Life sciences ☐ Behavioural & social sciences ☒ Ecological, evolutionary & environmental sciences

For a reference copy of the document with all sections, see nature.com/documents/nr-reporting-summary-flat.pdf

Ecological, evolutionary & environmental sciences study design

All studies must disclose on these points even when the disclosure is negative.

Study description	This was a retrospective analysis of previously collected samples from multiple African countries.
Research sample	The primary samples were collected as dried blood spots during the 2013 DHS survey done in the DRC. This was a study of 18,171 households with details described here: https://dhsprogram.com/what-we-do/survey/survey-display-421.cfm.<br><br>Outside of the DRC, samples were sourced from previously collected sample sets. Samples from Ghana, Tanzania and Uganda were part of previously reported studies and the references to those studies are included in the manuscript. Samples from Zambia were collected as part of ongoing NIH ICEMR activities in that country and have not been reported on previously. These were RDT positive individuals from a community survey of all ages in Nchelenge District in northeast Zambia on the border with the DRC.
Sampling strategy	Sampling strategy for the DHS is described in their documentation. This is a population representative nationwide survey.<br><br>Other samples are convenience samples selected to be temporally similar to the DHS.
Data collection	Data collection (sequencing) was done at Brown University by PM, MD and TF. DNA on 96 well plates were used in the capture in a 96 well format. Barcoding files based on these maps were generated by PM and transfered to OA for demultiplexing of sequencing runs. Demultiplexed data was analyzed by codes developed by OA. All processes were automated to reduce error.
Timing and spatial scale	Timing and spatial details of DHS samples are available at their website: https://dhsprogram.com/what-we-do/survey/surveydisplay-421.cfm. Samples were collected between August 2013 and February 2014.<br><br>Timing of the Tanzania, Ghana and Uganda sampling is published in the respective manuscripts.<br>Zambian samples were collected May to July of 2013 and were confined to a catchment area for Nchelenge.
Data exclusions	Data was only excluded for not making it through the sequence filtering pipeline described in the paper. Within each sample, variants were dropped if they had a Phred-scaled quality score of <20. Across samples, variant sites were dropped if they were observed only in one sample, or if they had a total UMI count of less than 5 across all samples. Sites were restricted to SNPs, and in the case of the genome-wide panel these were filtered to the pre-designed biallelic target SNP sites. Any variant that was represented by a single UMI in a sample, or that had a within-sample allele frequency (WSAF = UMI count/coverage) less than 1%, was eliminated. Any site that was invariant across the entire dataset after this procedure was dropped. Samples were assessed for quality in terms of the proportion of low-coverage sites, where low-coverage was defined as fewer than 10 supporting UMIs. Samples with >50% low-coverage loci were dropped. Variant sites were then assessed by the same means in terms of the proportion of low-coverage samples, and sites with >50% low-coverage samples were dropped. Samples were then combined with metadata, including geographic information, and were only retained if there were at least 10 samples in a given country. Exclusions were determined by the distribution of the data.

Reproducibility	The original analysis was carried out once. We confirmed the findings by repeating the analysis masking the SNPs around drug resistance alleles to ensure our findings of the cline in PC1 were valid and held without confounding from the drug resistance regions. We found the same pattern in this second analysis, supporting our initial conclusions.
Randomization	This is a population study and thus samples were analyzed as one group for the majority of analyses. Exceptions to this include:<br>1. For analysis of drug resistance haplotypes: This requires the use of monoclonal infections. We used the data from the REAL McCOIL analysis to limit this analysis to only these samples. We overlapped the drug resistance MIP data and SNP MIP dtat to determine a set of 143 samples where we were sure the sample was monoclonal and we had drug resistance allele data.<br>2. Extended haplotype analysis: The same restrictions as #1 were needed for the extended haplotype analysis and therefore the same subset of samples were used.
Blinding	All sequencing variant calling was carried out by OA to make a final dataset for filtering. RV, NB and OW were all provided this set to link to other data and continue analysis.<br><br>As this was a fixed sample and dataset, blinding was not necessary for this study.

Did the study involve field work? ☐ Yes ☒ No

Reporting for specific materials, systems and methods

We require information from authors about some types of materials, experimental systems and methods used in many studies. Here, indicate whether each material, system or method listed is relevant to your study. If you are not sure if a list item applies to your research, read the appropriate section before selecting a response.

Materials & experimental systems

n/a	Involved in the study
☒	☐ Antibodies
☒	☐ Eukaryotic cell lines
☒	☐ Palaeontology
☒	☐ Animals and other organisms
☐	☒ Human research participants
☒	☐ Clinical data

Methods

n/a	Involved in the study
☒	☐ ChIP-seq
☒	☐ Flow cytometry
☒	☐ MRI-based neuroimaging

Human research participants

Policy information about studies involving human research participants

Population characteristics	This was a retrospective analysis of de-identified human samples. Thus, this is technically not human subjects research, but uses human material.
Recruitment	Not applicable
Ethics oversight	Original ethical oversight was done by the studies which provided samples. All participants in those studies provided informed consent with the work performed here falling under the scope of consent. IRBs for the secondary users (e.g. UNC) deemed the work to be non-human subjects.

Note that full information on the approval of the study protocol must also be provided in the manuscript.
