# Supplementary material for: The impact of antimalarial resistance on the genetic structure of Plasmodium falciparum in the DRC
Source: Nat Commun. 2020 Apr 30;11:2107. doi: 10.1038/s41467-020-15779-8 (PMC7192906; doi:10.1038/s41467-020-15779-8)
Supplement: Supplementary file 4 — Description of Additional Supplementary Files [file 41467_2020_15779_MOESM4_ESM.pdf]

Description of Additional Supplementary Files

File Name: Supplementary Data 1
Description: Genomes used for MIP Design- The sample name (Sample), short read archive accession number (Run Accession), and the country of origin for the genomes used in the molecular inversion barcode design.

File Name: Supplementary Data 2
Description: Prevalence of Antimalarial Resistance Mutations- The prevalence of antimalarial resistance mutations detected by the MIPs is shown for each geographic location.

File Name: Supplementary Data 3
Description: Design of SNP MIPs- The design of each MIP used in the SNP barcode is provided.
